# Feasibility of model-based Roentgen Stereophotogrammetric Analysis to evaluate early migration of the trapeziometacarpal joint prosthesis

**DOI:** 10.1186/s12891-015-0747-3

**Published:** 2015-10-14

**Authors:** E.M. Ooms, B. ten Brinke, N.M.C. Mathijssen, I.F. Blom, R.L.M. Deijkers, G.A. Kraan

**Affiliations:** Centre for Orthopaedic Surgery, OCON, Hand and Wrist unit, Hengelo, The Netherlands; Department of Orthopaedic Surgery, Reinier de Graaf Groep, Delft, The Netherlands; Departments of Radiology and Orthopaedic Surgery, Reinier de Graaf Groep, P.O. Box 5011, 2600 GA Delft, The Netherlands; Department of Orthopaedic Surgery, Haga Ziekenhuis, Den Haag, The Netherlands

**Keywords:** Roentgen Stereophotogrammetric Analysis (RSA), Thumb arthrosis, TMC joint arthroplasty, Micromotion, Early migration

## Abstract

**Background:**

The purpose of this study was to determine the feasibility of Roentgen Stereophotogrammetric Analysis (RSA) in total joint arthroplasty of the trapeziometacarpal (TMC) joint of the thumb.

**Methods:**

In five cadaveric hands the TMC-joint was replaced by the Surface Replacement Trapeziometacarpal prosthesis (SR™ TMC prosthesis; Avanta, San Diego, CA) and tantalum beads of 0.8 mm were implanted for RSA. RSA radiographs in two directions were made in ten positions to calculate the measurement error. Migration values from zero are indicative for the measurement error. The number of detected markers was recorded.

**Results:**

The accuracy analysis showed that for the translations the mean measurement error varied between 0.003 mm (SD 0.057) and 0.055 mm (SD 0.133). For the rotations values ranged from 0.034° (SD 1.759) to 0.502° (SD 1.617).

**Conclusions:**

RSA analysis of the SR™ TMC prosthesis is feasible. The measurement error is good for the translations but high for the rotations. The latter is due to the close position of the markers relative to each other. Level of evidence III.

## Background

**Fig. 1:**
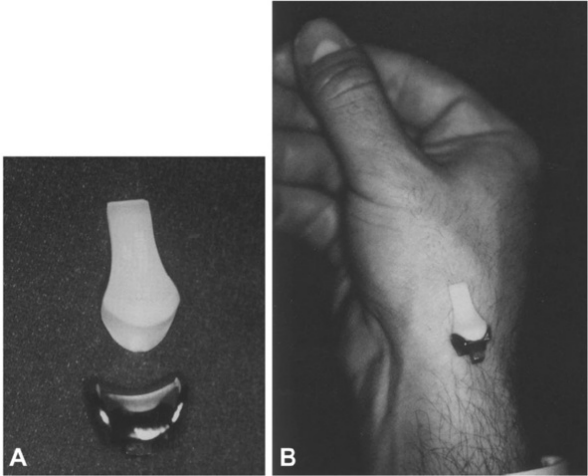
SR TMC prosthesis in front of TMC joint

Osteoarthritis of the trapeziometacarpal joint (TMC joint) is a disabling disease. The prevalence of trapeziometacarpal osteoarthritis (OA) is estimated to be 2.2 % in women and 0.62 % in men. A high prevalence is found in older women (70–74 years) with an estimate of 5.3 % [1]. Restoration of thumb function with a pain free, stable and mobile joint while preserving strength is the main goal of surgical treatment [2, 3]. Several prosthesis designs for TMC joint replacement have been used with variable success rates, however early failure remains an important issue [4, 5]. These failures are mainly due to aseptic loosening caused by implant instability [6]. A relatively new prosthesis design, the SR™ TMC prosthesis (Avanta, San Diego, CA), is a resurfacing joint replacement that closely duplicates the anatomy of the articular surfaces of the first metacarpal and trapezium (Fig. 1) [7].

This prosthesis might perform better in terms of survival, which is highly dependent on implant stability [8]. Clinical reports of the SR™ TMC prosthesis however show loosening rates from zero to 55 % [5, 9, 10].

In all studies concerning TMC joint replacement, aseptic loosening is scored when radiolucency or gross displacement is seen while comparing subsequent x-rays [4, 5, 11, 12]. However, this method is far from accurate. In larger joints, implant stability can be assessed with high accuracy using Roentgen Stereophotogrammatric Analysis (RSA) [13]. The usefulness of RSA in larger joints as the knee and the hip has been shown in two recent systematic reviews [14, 15] and RSA has become the gold standard for research in prosthesis survival [16]. Hansen et al. described the use of RSA in the trapeziometacarpal joint in a phantom study [17]. Their research showed that RSA might be clinically useful for detection of loosening of the prosthesis up to two years [18]. However, since only one phantom study and one clinical study have been performed using RSA in the TMC joint, we may state that there is little experience in this field. Furthermore, only the cemented metacarpal cup (DLC cup, Small Bone Innovations Inc.) and the Elektra trapezium screw cup (Small Bone Innovations Inc) were analysed by Hansen et al. and not the saddle formed SR™TMC joint prosthesis as used in this study. Moreover, accuracy of rotation values was poor in the research that has been done so far [17, 18].

Therefore, before new clinical RSA studies should be performed, we first performed a RSA cadaver study using the SR™TMC joint.

RSA of the TMC joint can be challenging because of the limited surgical exposure and the small available bone stock for placement of RSA beads. The purpose of this study therefore was to determine if RSA is feasible in TMC joint replacement using the SR™ TMC prosthesis and if so, what the measurement error is when using this technique.

## Methods

**Fig. 2:**
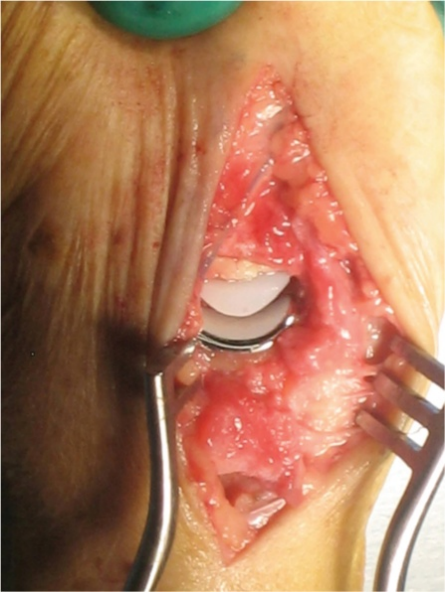
Implanted SR TMC prosthesis

In five cadaveric hands the TMC joint was replaced by the SR™ TMC prosthesis according to the standard implantation technique as described by the manufacturer (Avanta orthopaedics, San Diego, USA) (Fig. 2).

Tantalum beads of 0.8 mm were implanted in the trapezium and first metacarpal bone without the need for extension of the skin incision or extending the standard surgical exposure. In general, in the trapezium three beads were implanted via the 1 mm drilled hole for the prosthetic peg and two more were inserted through the exposed radial cortex. In the first metacarpal two beads were placed in metaphyseal bone as distal as possible via the reamed intramedullary cavity. Additionally one more bead was inserted in the ulnar trabecular bone of the metacarpal base and one or two beads secured in the exposed radial cortex. The metacarpal prosthesis component was provided with three or four 0.5 mm beads, two at the tip and one or two at the base of the component. Insertion of the beads was performed with a combined instrument of a 0.7 or 1.1 gauge i.v. needle and the trocart of a 1.1 gauge spinal needle.

**Fig. 3:**
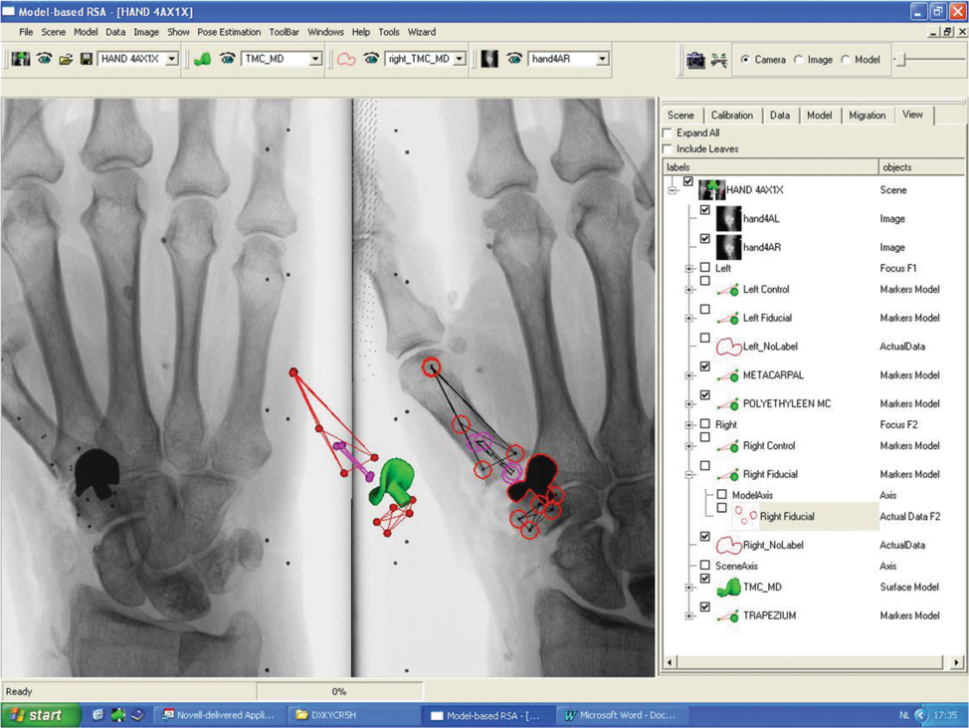
Model based RSA scene of the implanted SR TMC prosthesis. 3D reconstruction image in the centre of the figure shows the position of the trapezium component, markers in the polyethylene metacarpal component and the markers in inserted in bone (first metacarpal and trapezium). (*if in colour print)*: Model based RSA scene of the implanted SR TMC prosthesis. 3D reconstruction image in the centre of the figure shows the position of the trapezium component (green), markers in the polyethylene metacarpal component (purple) and the markers in inserted in bone (first metacarpal and trapezium, red)

A reversed engineered three-dimensional surface model of the trapezium component of the SR™ TMC prosthesis was prepared for model-based RSA analysis (Introtech, Nuenen, The Netherlands) [19]. After the surgical procedure, RSA radiographs were made using a carbon fibre calibration box (Medis specials, Leiden, The Netherlands) and two synchronized roentgen tubes. RSA radiographs were performed of all hands in two commonly used positions for imaging of the TMC joint (Robert view and lateral view). The number of visually detected markers for each bone/implant was recorded. Of each hand, ten pairs of RSA radiographs were made. After each radiograph, the hand was replaced and rotated a few degrees. The radiographs were imported in a software program for model-based RSA (Model-based RSA 3.11, Medis specials, Leiden, The Netherlands) and the ‘migration’ of the prosthesis between the RSA radiographs was calculated (Fig. 3). All markers (i.e. fiducial, control and intra-ossal) and the prosthesis were marked manually in both planes. Paired migrations were performed to calculate the ‘migration’ between all ten positions of each hand. To obtain the accuracy of the performed technique, mean errors and standard deviations were calculated for all translations and rotations.

The study protocol have been assessed by the regional Medical Ethical Committee (METC Zuidwest Holland) and no ethical approval was necessary, since this study did not fall under the scope of the Medical Research Involving Human Subjects Act.

## Results

For the metacarpal bone, all beads were visible in all positions and both RSA radiographs. For the polyethylene metacarpal prosthesis component one of the five specimen had an over projection of the proximal bead by the metal trapezium prosthesis component. If five beads were used in the trapezium, at least three beads were visible in all positions. The accuracy analysis showed that for the translations the measurement error varied between 0.003 mm (SD 0.057) and 0.055 mm (SD 0.133). For the rotations values ranged from 0.034° (SD 1.759) to 0.502° (SD 1.617). The accuracy analysis is presented in Table 1.

**Table 1:** Measurement errors of model-based RSA of the metacarpal and Trapezium component of the SR™TMC prosthesis after repeated measurements of five cadaveric hands in ten different positions. X,Y,Z: translations along the x-axis (medial-lateral), y-axis (distal-proximal) and z-axis (posterior-anterior). Rx,Ry,Rz: rotations around the x-axis (flexion-extension), y-axis (internal-external) and z-axis (abduction-adduction)

Metacarpal Component	X (mm)	Y (mm)	Z (mm)	Rx (°)	Ry (°)	Rz (°)
Average	0,006	-0,003	0,055	-0,034	0,502	0,043
SD	0,098	0,181	0,133	1,759	1,617	1,069
Min	-0,156	-0,272	-0,150	-3,495	-1,699	-2,242
Max	0,152	0,319	0,225	2,958	3,295	0,830
Trapezium Component	X (mm)	Y (mm)	Z (mm)	Rx (°)	Ry (°)	Rz (°)
Average	0,025	0,003	-0,034	-0,148	-0,045	-0,474
SD	0,093	0,057	0,082	0,749	0,762	1,085
Min	-0,057	-0,077	-0,117	-1,272	-0,703	-2,666
Max	0,253	0,104	0,142	1,157	1,830	1,045

## Discussion

This is the first study in which the accuracy of RSA was measured and analysed in TMC joint arthroplasty using the SR™ TMC prosthesis. In surgical procedures that do not occur frequently, as the trapeziometacarpal joint replacement, the high accuracy of RSA is essential whereas only small patient cohorts can be achieved to evaluate the effect on prosthetic fixation due to changes in implant design, addition of coatings, surgical placement technique or new bone cements [20, 21]. It is suggested by Valstar et al. that the validation of the accuracy of RSA systems can be carried out with high accuracy [16]. Therefore, we performed this phantom study on cadaveric hands. The results of this study can be used for a clinical study on the TMC joint. We conclude that with the amount and the different diameters of the tantalum beads, RSA radiographs can be made that are accurate and easy to interpret. The bead placement does not influence the extent of the surgical procedure, although a somewhat longer operation time is inevitable. The reported accuracy of RSA in literature (expressed as the standard deviations of repeated measurements) ranges between 0.08 and 0.22 mm for translations and between 0.15° and 0.52° for rotations [22]. Regarding the accuracy of RSA in the TMC joint, standard deviations varied between 0.03 and 0.77 mm for translations and 0.40°–13.08° for rotations in a phantom study [17]. In a clinical study the highest standard deviations were 0.25 mm for translations and 12.69° for rotations [18]. Measured accuracy in this study is comparable to these accuracy results, with respect to the translation. Standard deviations of rotation values were also high (highest SD 1.759°)., but not as high as in the phantom study of Hansen et al. This could be due to the asymmetric shape of the SR™TMC prosthesis in contrast to the symmetry along the Y-axis of the Elektra HA stem.

The low accuracy of rotation values in our study is expected to be due to the close position of the markers relative to each other in the first metacarpal and the metacarpal prosthesis component. Further, the high measurement errors of rotations could be the result of selecting different sets of beads during the analysis of RSA radiographs from different positions, since not all five beads were visible in each direction.

To decrease the measurement error in future (clinical) studies, the distance between the markers should be enlarged. Beside, beads should be placed in a triangular fashion in the metacarpal component, instead of four in a rectangle.

In joint replacement surgery aseptic mechanical loosening is the main reason for long-term revision. It might be caused by wear particles from the articular surface that causes osteolytic activity around the prosthesis. Another cause of mechanical loosening could be insufficient bone-prosthesis fixation or high stresses on the bone-prosthesis interface due to a bad design of the implant [4, 6, 23, 24]. Aseptic mechanical loosening starts with severe or ongoing migration, in the range of 0.2 to 1.0 mm, of the prosthesis relative to the bone. Since loosening of a prosthesis starts with migration, knowledge on migration is important as it could predict future loosening or gain more insight about the fixation [25]. With this insight the design of TMC prosthesis could be further improved.

## Conclusions

RSA analysis of the SR™ TMC prosthesis is feasible. The measurement error is good for the translations but high for the rotations. The latter is due to the close position of the markers relative to each other.

## Abbreviations

DLC: Diamond-like-carbon
HA: Hydroxyapatite
METC: Medisch Ethische Toetsingscommissie (Medical Ethical Committee)
OA: Osteoarthritis
RSA: Roentgen Stereophotogrammetric Analysis
SD: Standard Deviation
SR TMC: Surface Replacement Trapeziometacarpal
TMC: Trapeziometacarpal
